# High quality process of care increases one-year survival after acute myocardial infarction (AMI): A cohort study in Italy

**DOI:** 10.1371/journal.pone.0212398

**Published:** 2019-02-20

**Authors:** Martina Ventura, Valeria Belleudi, Paolo Sciattella, Riccardo Di Domenicantonio, Mirko Di Martino, Nera Agabiti, Marina Davoli, Danilo Fusco

**Affiliations:** 1 Department of Epidemiology of Lazio Regional Health Service, Rome, Italy; 2 Department of Statistical Sciences, “Sapienza” University of Rome, Rome, Italy; Azienda Ospedaliero Universitaria Careggi, ITALY

## Abstract

**Background:**

The relationship between guideline adherence and outcomes in patients with acute myocardial infarction (AMI) has been widely investigated considering the emergency, acute, post-acute phases separately, but the effectiveness of the whole care process is not known.

**Aim:**

The study aim was to evaluate the effect of the multicomponent continuum of care on 1-year survival after AMI.

**Methods:**

We conducted a cohort study selecting all incident cases of AMI from health information systems during 2011–2014 in the Lazio region. Patients’ clinical history was defined by retrieving previous hospitalizations and drugs prescriptions. For each subject the probability to reach the hospital and the conditional probabilities to survive to 30 days from admission and to 31–365 days post discharge were estimated through multivariate logistic models. The 1-year survival probability was calculated as the product of the three probabilities. Quality of care indicators were identified in terms of emergency timeliness (time between residence and the nearest hospital), hospital performance in treatment of acute phase (number/timeliness of PCI on STEMI) and drug therapy in post-acute phase (number of drugs among antiplatelet, β-blockers, ACE inhibitors/ARBs, statins). The 1-year survival Probability Ratio (PR) and its Bootstrap Confidence Intervals (BCI) between who were exposed to the highest level of quality of care (timeliness<10', hospitalization in high performance hospital, complete drug therapy) and who exposed to the worst (timeliness≥10', hospitalization in low performance hospital, suboptimal drug therapy) were calculated for a *mean-severity* patient and varying gender and age. PRs for patients with diabetes and COPD were also evaluated.

**Results:**

We identified 38,517 incident cases of AMI. The out-of-hospital mortality was 27.6%. Among the people arrived in hospital, 42.9% had a hospitalization for STEMI with 11.1% of mortality in acute phase and 5.4% in post-acute phase. For a *mean-severity* patient the PR was 1.19 (BCI 1.14–1.24). The ratio did not change by gender, while it moved from 1.06 (BCI 1.05–1.08) for age<65 years to 1.62 (BCI 1.45–1.80) for age >85 years. For patients with diabetes and COPD a slight increase in PRs was also observed.

**Conclusions:**

The 1-year survival probability post AMI depends strongly on the quality of the whole multicomponent continuum of care. Improving the performance in the different phases, taking into account the relationship among these, can lead to considerable saving of lives, in particular for the elderly and for subjects with chronic diseases.

## Introduction

According to the Global Burden of Diseases estimates, ischemic heart diseases still remains the leading cause of premature death worldwide and in Italy [1]. In particular, each year more than 7 million people experience myocardial infarction with one-year mortality rates in the range of 10%, varying with patient characteristics [2–3].

The management of acute myocardial infarction (AMI) is built on clinical evidence drawn from many studies undertaken over the past three decades. The evolution in clinical practice has substantially reduced mortality and morbidity associated with the condition. [3]

The first few hours after symptom onset are the most critical in AMI and over 50% of cardiac deaths occur within the first 30 minutes, when the patient has not yet reached the hospital [4]. Several studies focused on the effect the overall system delay on adverse outcomes, including all components of delay modifiable by the health care system, both pre-hospital and door-to-balloon time [5–8]. Thus, reducing the interval between symptom onset and the hospital arrival (emergency phase) as well as receiving a timely and appropriate in-hospital medical treatment, are crucial factors in the survival of these patients [9, 10]. In particular, for patients with ST-elevation myocardial infarctions (STEMI), the European Society of Cardiology and the American College of Cardiology/American Heart Association recommend primary percutaneous coronary intervention (PCI) within 90 minutes of first medical contact and a total ischemic time, from symptom onset to reperfusion, within 120 minutes [11,12].

The relationship between timely PCI with respect to both short-term and long-term mortality has been widely studied [13–18]. However, a recent study showed that the protective effect of a timely reperfusion of STEMI is reduced with increasing travel time from home to hospital [8], suggesting a strong effect of the quality of care on survival. Regarding long-term survival after AMI, several aspects following the initial acute phase should be considered as life behaviors and the adherence to drug therapy [19]. Platelet aggregation inhibitors (antiplatelets), β-blocking agents (β-blockers), agents acting on the renin-angiotensin system (ACEI angiotensin receptor blockers) and 3-hydroxy-3-methylglutaryl-coenzyme A (HMG-CoA) reductase inhibitors (statins) are well established therapeutic strategies for post-AMI secondary prevention [20, 21]. There is large evidence of suboptimal adherence to evidence based polytherapy in the post-acute phase for AMI patients [22–24], despite the beneficial effect on prognosis has been clearly shown [25–27].

In our Region in the last ten years on behalf of the Outcomes Evaluation Program in Lazio Region a number of “process of care indicators” related to the management and treatment of AMI are calculated, on the basis of a standardized methodology, periodically diffused on a website and used in audit and feedback multidisciplinary programs to improve clinical practice [28,29]. According to both clinical guidelines and regional AMI committee recommendations, these process indicators identify well recognized critical points of the AMI care, starting from the emergency to the post-acute phase [11,12,28–31].

They include the proportion of PCIs performed on STEMI patients, the proportion of PCI executed within 90 minutes among all reperfusion procedures executed within 12 hours, and the adherence to evidence-based drug treatment in the 30 days after discharge. Moreover, a shorter travel time to hospital has been shown to improve the outcomes of AMI care in our Region [8], as observed in other contests [32].

In recent years, the efforts of health care systems have been focused separately on primary prevention programs, on reducing the delay in the management of the patient, and on improving the adherence to EB therapy. Actually, the care of AMI patients is a continuous lifelong process in which all the phases should not be considered in isolation but integrated. Exploring the whole multicomponent process of care (MPC) and understanding its effect on patient survival should be useful for health planning purposes. Moreover, the effect of the MPC is influenced by patient’s clinical profile [33–35]; therefore, the identification of subgroups of patients with particular needs could contribute to better target the interventions.

We hypothesized that AMI patients exposed to a high quality process of care along the complex multicomponent continuum of care would experience a better long-term prognosis. The aims of this study were to evaluate the effect of the whole process of care on 1-year survival and to analyze the role of gender, age and chronic diseases on the relationship between the quality of care and survival.

## Materials and methods

### Data sources

Data were collected using the health information systems (HIS) of the Lazio region of Italy. Information on hospitalizations, emergency visits, drug prescriptions and cause-specific mortality is available for each individual registered in the Health Care Assistance Registry (approximately 97% of residents), and have been integrated using a deterministic record-linkage procedure based on anonymous identification codes. In this way, we created a chronological, demographical, residential, clinical, health-related patient profile. We derived additional information on comorbidities and time to surgery through the regional Admission and Discharge Information System.

### Ethics

This study was carried out in full compliance with the current privacy laws. The Department of Epidemiology is legitimised by the Lazio Regional Committee in managing and analysing data from the regional health information systems for epidemiological purposes.

### Study cohort

The present retrospective cohort study was based on the population aged 18–100 years, living in the Lazio region of Italy and registered in the regional health system. Lazio is the central region of Italy with about 5,5 million inhabitants, corresponding to 10% of the Italian population.

AMI cohort was identified selecting all hospital admissions with a main diagnosis of AMI or main diagnosis of an AMI-related condition along with a secondary diagnosis [28], and deaths for ischemic heart disease (ICD-9 410–414) [8] between 1 January 2011 and 31 December 2014. Only incident cases were considered (index event): patients with a hospital admission for AMI in the previous 3 years were excluded from the analysis.

### Patient characteristics

For each patient, demographic and clinical characteristics were recorded in the index event. Moreover, patient clinical history was defined by retrieving specific conditions and procedures during hospitalizations or emergency visits in the previous 2 years, such as cancer, hypertension, hearth failure, arrhythmias, previous coronary angioplasty, other forms of ischemic heart diseases, chronic nephropathies [28]. The presence of diabetes and chronic obstructive pulmonary disease (COPD) was assessed in order to identify patients with a more complex clinical picture, using a validated algorithm based on ticket exemption, drug prescriptions and hospital admissions [36–38].

To better define the clinical profiles of the patients, the use of drugs in the 6 months prior to the index event was also evaluated: cardiac therapies, antiplatelet therapies, anticoagulants, antihypertensive drugs, diuretics, beta-blocking agents, calcium channel blockers, angiotensin-converting-enzyme inhibitors, and angiotensin II antagonists or statins.

### The multicomponent continuum of care process

The AMI care was considered as the process from the onset of symptoms to the secondary prevention post hospital discharge. Three phases were analyzed: the *emergency* phase, from the onset of symptoms to the arrival in an appropriate facility; the *acute* phase, from the arrival in hospital to the discharge; the *post-acute* phase, from the hospital discharge up to 1 year.

In each phase, we identified an exposure that measured the actual quality of care and took into account the specific level of intervention (individual or hospital) to be considered in order to promote the improvement of health assistance.

For the emergency phase, the travel time from home to the nearest emergency service was considered as exposure. After standardizing and geocoding patients and facilities addresses, the nearest hospital was assessed using a road network-based route analysis, that allowed to calculate the minimum driving travel time (in minutes) from patient address to hospital [8].

We classified exposure considering the median of the travel time’s empirical distribution (i.e. lower or higher than ten minutes).

In the acute phase, we considered patients with STEMI. At this stage, only hospital records were analyzed, making it possible to identify STEMIs through the reported ICD9-CM diagnoses. The exposure was based on the appropriateness and timeliness of the treatment and defined at hospital level. The hospital performance was described as the combination of two variables: the proportion of PCI performed on STEMI patients and the proportion of PCI executed within 90 minutes among all reperfusion procedures executed within 12 hours. Hospitals in the highest quartile of the distribution of both variables were classified as “high performance” and those in the lowest quartiles as “low performance”. All other facilities were classified as “medium performance”.

The exposure of the post-acute phase was based on the adherence to the EB drug treatment in the 30 days after discharge (intention to treat approach). Information about prescriptions of antiplatelets (ATC: B01AC04, B01AC05, B01AC06), β-blockers (ATC: C07), ACEI/ARBs (ATC: C09) and statins (ATC: C10AA) were retrieved for all patients. The number of EB drugs was evaluated (at most 2, 3 or 4).

Patients who died within 30 days after discharge or those with a hospital stay longer than 28 days were excluded in order to reduce misclassification and heterogeneity of the exposure in the post-acute phase.

By combining the phase-specific exposures, we obtained 18 different possible MPC scenarios. In particular, the best and the worst scenarios were respectively defined as:

- travel time lower than 10 minutes, admission in a high performance hospital, complete EB drug therapy;
- travel time of 10 minutes or more, admission in a low performance hospital, less than three EB drugs.

### Outcomes

Three different outcomes post AMI were considered: the probability of reaching the nearest hospital alive; the probability of 30 days survival, given the survival to the emergency phase; the probability of 31–365 days survival, given the survival to the acute phase. The overall 1-year survival was defined as a combination of three outcomes.

### Statistical analysis

As described in detail previously [39], we used multivariate logistic regression models to assess the effect of MPC on the three outcomes, accounting for demographic characteristics, comorbidities and co-medications of the patients. Among all factors potentially associated with the outcome, age and gender were considered to be a priori risk factors, the other factors were selected using a stepwise bootstrap procedure. Using this approach, 1000 replicated bootstrap samples were selected from the original cohort. A bootstrap sample is a sample of the same size as the original dataset chosen with replacement. Thus, a given subject in the original cohort may be selected multiple times, only once, or not at all in a specific bootstrap sample. A stepwise procedure, using thresholds of p  =  0.05 for variable selection and elimination, was applied to each replicated sample, and only the factors selected in at least 50% of the procedures were included in the final models.

In order to take into account the variability of survival between hospitals, a multilevel approach was applied in the acute and post-acute phase considering hospital of admission as a second level unit.

**Fig 1:**
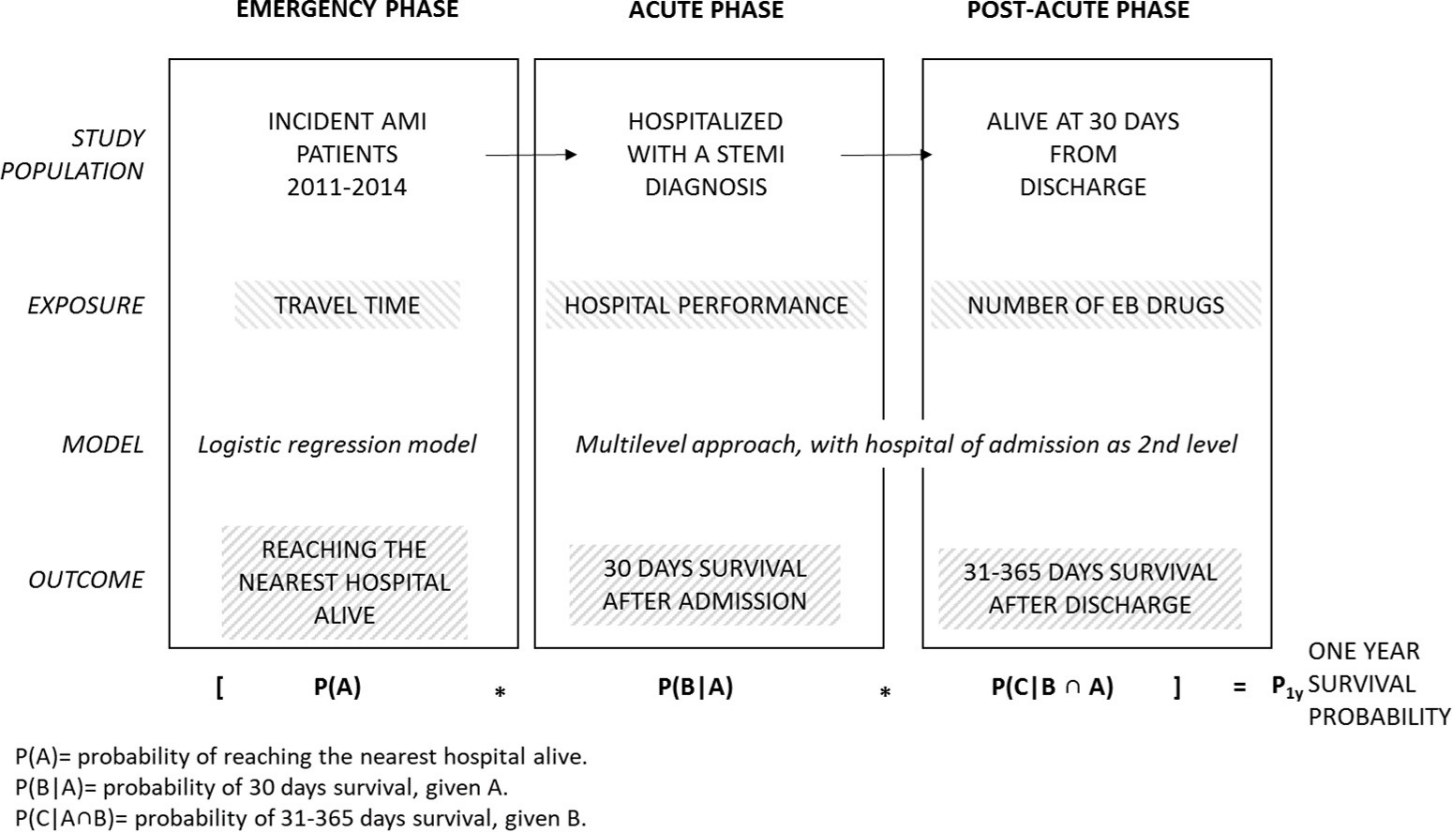
Models details.

For each phase of the MPC the probability of survival was estimated considering a *mean-severity* patient, i.e. a patient with the same distribution of age, sex and mortality risk factors as observed in the cohort. Because the survival in each phase is conditioned to the survival in the previous phases, the overall 1-year survival probability was calculated as product of the three probabilities (Fig 1).

To assess the effectiveness of the MPC, the 1-year probability ratio (PR) between the best and the worst scenarios was calculated, and its Bootstrap Confidence Intervals (95% BCI) were estimated.

Furthermore, to estimate the potential saving of lives due to the MPC, for each phase the survival probability estimated for the best MPC was applied to the patients who experienced the worst MPC.

Finally, in order to evaluate the potential effect of demographic or clinical factors on the relationship among care-pathways and survival, the PRs were calculated by gender, age classes and considering patients with chronic conditions.

### Sensitivity analysis

To assess the robustness of our results, some sensitivities analyses were performed. To evaluate the quality of exposure in the emergency phase the association between travel time and instantaneous survival was estimated in a subgroup of patients aged 75 years or more. The association between drug therapy and survival was examined using a Cox regression model with mixed effects—frailty model—in order to take into account the length of survival in the post-acute phase. Lastly, to evaluate the quality of exposure in the post-acute phase, we replicated the main analysis on the subgroup of new drug users.

## Results

Between 2011 and 2014, 38,517 incident cases of AMI were identified in Lazio region. They were mainly men (61.8%) and older than 65 years (mean age 73 years). Diabetes and COPD were found in 29% and 21% of the cohort, respectively.

**Fig 2:**
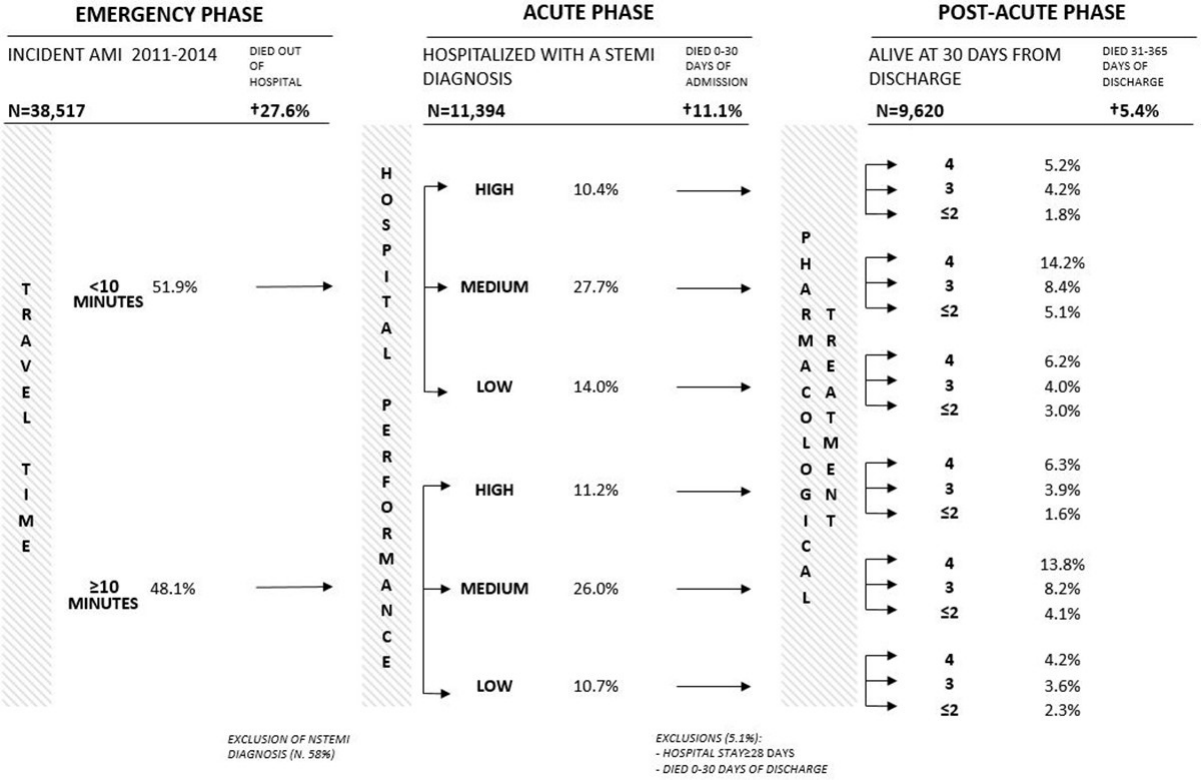
Process of care for AMI patients.

During the emergency phase, 27.6% of AMI died before reaching a hospital. Among those who survived 11,394 were admitted in a regional hospital with a STEMI diagnosis (42%) and of those, 11.1% died within 30 days of admission. In the post-acute phase 9,620 patients were analyzed and 5.4% died within 31–365 days post discharge (Fig 2).

As observed in Fig 2, eighteen different MPCs were identified by combining the different exposures. About half of the study population could reach a hospital with hemodynamic in less than ten minutes. In the acute phase, 21.6% (of which 10.4% with travel time lower than 10 minutes) and 24.7% was admitted respectively in high and low performance hospital. Considering the patients survived to the acute phase, 49.9% followed a 4-drugs EB therapy whilst at most 2 drugs were found in 18.0% of the cases. Thus, we found that patients experienced the best MPC in 5.2% of cases, whereas they followed the worst one in 2.3%.

Table 1 shows the determinants of survival in each phase of the AMI care In all the phases, no difference was found for gender, while a decreasing survival was observed with an increase in age. Patients with diabetes had a higher probability of survival to the emergency phase (OR = 1.44 p = < .0001), but a lower survival was found for these patients in the acute and post-acute phases (OR = 0.71 p = < .0001 and OR = 0.69 p = < .0001, respectively). For COPD patients a lower survival was observed in all the phases. Having a travel time of ten minutes or more was associated with a lower probability of reaching the hospital alive (OR = 0.90 p = < .0001). The effect of the hospital performance was found in the acute and post-acute phases: patients hospitalized in high performance facilities had a higher 30 days survival (OR = 1.49 p = 0.018), whereas those admitted in low performance hospitals had a lower long-term survival (OR = 0.62 p = 0.002). Lastly, to be adherent to the EB pharmacological treatment in the post-acute phase was associated with an increasing survival: patients undergoing a complete therapy (4 drugs) had a more than twofold probability of 1-year survival than those with at most 2 drugs (OR = 2.62 p = < .0001).

**Table 1:** Association between process of care and survival.

		EMERGENCY				ACUTE					POST ACUTE		
*Patients*	* *	38517				11394				9620			
		Reaching the hospital alive 72.4%				30 days survival 88.9%				31–365 days survival 94.6%			
** **	** **	**%**	OR adj*	**95%CI**	**p-value**	**%**	OR adj*	**95%CI**	**p-value**	**%**	OR adj*	**95%CI**	**p-value**
**Gender**	**Men**	61.8	1			69.4	1			72.3	1		
	**Women**	38.2	1.03	0.97–1.08	0.360	30.6	0.89	0.77–1.01	0.080	27.7	0.96	0.78–1.19	0.705
**Age classes**	**<65**	27.5	1			42.8	1			48.4	1		
	**65–74**	21.0	0.61	0.56–0.66	< .0001	24.0	0.37	0.29–0.46	< .0001	24.7	0.44	0.31–0.61	< .0001
	**75–84**	27.7	0.37	0.34–0.40	< .0001	21.8	0.18	0.15–0.23	< .0001	19.1	0.24	0.17–0.33	< .0001
	**85+**	23.9	0.15	0.14–0.16	< .0001	11.4	0.08	0.07–0.10	< .0001	7.8	0.09	0.06–0.12	< .0001
**Diabetes**	** **	29.0	1.44	1.35–1.52	< .0001	24.4	0.71	0.62–0.81	< .0001	22.2	0.69	0.56–0.86	0.001
**COPD**	** **	21.3	0.91	0.86–0.97	0.003	14.4	0.89	0.76–1.04	0.143	12.6	0.80	0.63–1.02	0.076
**Travel time** **to hospital**	**<10**	51.9	1			52.1	1			72.3	1		
	**≥10**	48.1	0.90	0.86–0.94	< .0001	47.9	0.97	0.85–1.10	0.615	27.7	1.01	0.83–1.23	0.905
**Hospital** **performance**	**Medium**					53.7	1			53.7	1		
	**High**					21.6	1.49	1.08–2.07	0.018	23.0	1.00	0.70–1.44	0.984
	**Low**					24.7	0.94	0.73–1.21	0.617	23.3	0.62	0.47–0.82	0.002
** **	** **												
**Pharmacological** **treatment**	**≤2**									18.0	1.00		
** **	**3**									32.1	1.58	1.24–2.01	0.001
** **	**4**									49.9	2.62	2.03–3.38	< .0001

* Odds Ratio adjusted for sex, age, care pathway, chronic conditions, comorbidities and previous drug use.

**Fig 3:**
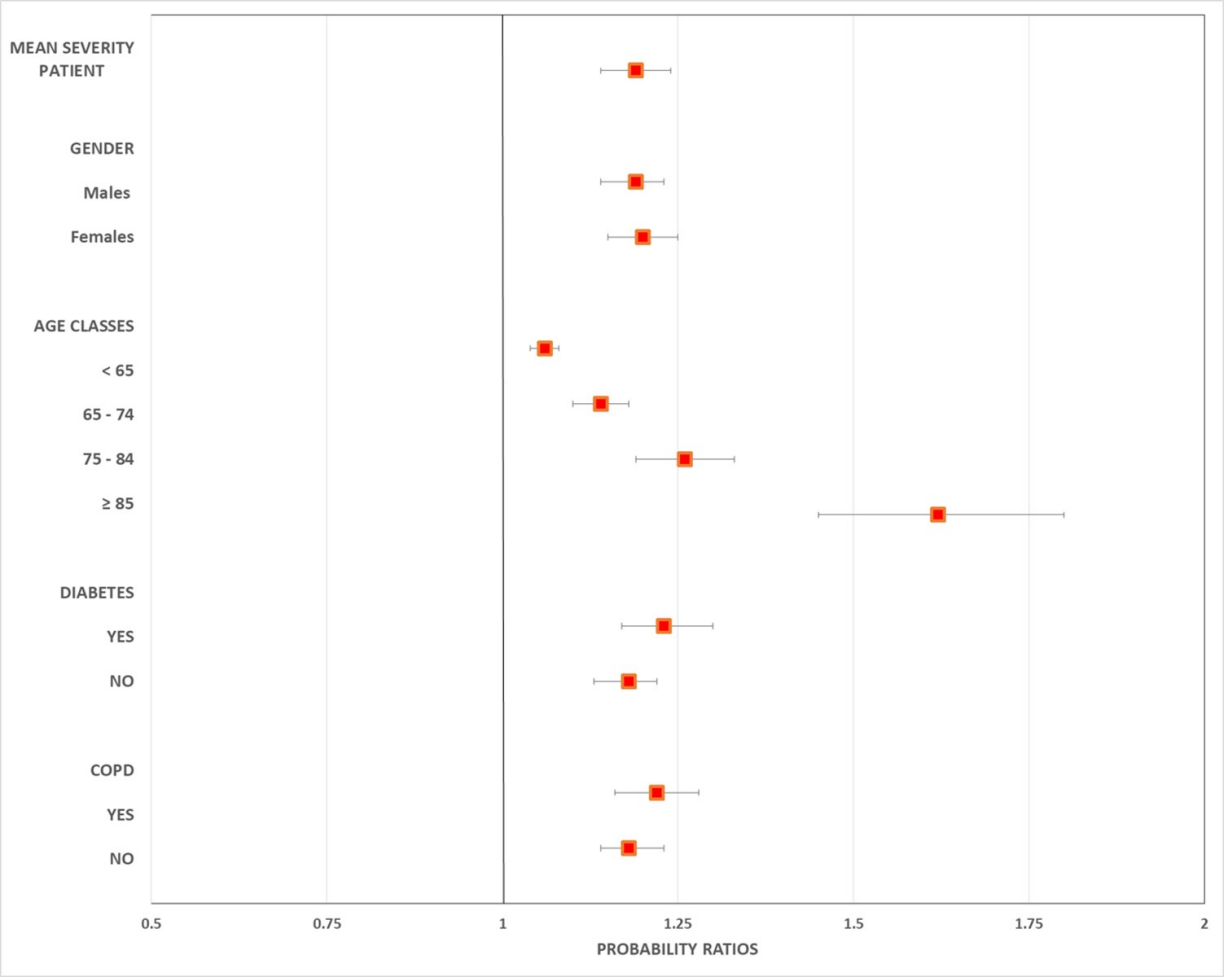
Probability ratios (PRs) between patients undertaking the best and the worst process of care–*mean-severity* patient, age, gender and chronic conditions.

Considering a *mean-severity* patient, the overall 1-year survival PR following the best MPC was 1.19 (BCI 1.14–1.24) as compared with the worst scenario. Applying the survival probabilities estimated for the best MPC to the patients who experienced the worst MPC we calculated a potential saving of about 400 lives. The PRs did not change by gender, whereas a strong increase was observed by age: it moved from 1.06 (BCI 1.05–1.08) for the youngest patients, to 1.62 (BCI 1.45–1.80) for age >85 years. For patients with diabetes and COPD a slight increase in PRs was found (Fig 3).

The sensitivity analysis confirmed the main results. In particular, in the emergency phase, patients aged at least 75 years with a travel time of ten minutes or more had a lower probability of reaching the hospital alive (OR = 0.94 p = 0.044). The higher survival associated with a complete EB pharmacological treatment in the post-acute phase was observed both when we took into account the length of survival, and when we considered the subgroup of new drug users.

## Discussion

In this study, we found that survival after AMI strongly depends on the quality of the whole multicomponent process of care: patients who experienced the best care, in terms of timeliness in the emergency, of admission in a high-performance facility, and of adherence to EB drug treatment, had a higher 1-year survival. If all patients followed the best care a considerable saving in lives would be observed.

As previously observed, in ST-elevation myocardial infarctions (STEMI) the majority of deaths occur during the first hours after symptom onset, and the beneficial effects of reperfusion treatment are critically time dependent [6, 40, 41]. De Luca et al., found that every minute of delay in treatment of patient with STEMI does affect 1-year mortality, with an increase of 7.5% for each 30-minute delay [42].

Several important time intervals need to be considered in the treatment of STEMI patients [43]. In the emergency phase, we focused on the relation between the time from home to the nearest emergency service and out of hospital mortality, and found that a higher travel time decreased the probability of reaching the hospital alive. Wei et al. reported a similar result for patients with their first AMI in Scotland, suggesting that distance from home to hospital may predict mortality outcome [44]. Moreover, in Lazio region, it has been demonstrated that travel time is a reliable proxy of pre-hospital system delay and a strong predictor of 30 days mortality after timely execution of PCI for STEMI [8].

The second time interval that we considered was the in-hospital time for patients with STEMI who reached the hospital alive. Health care systems are expected to treat those patients with PCI within 90 or 120 minutes from the first medical contact depending on whether they arrive in a PCI-capable or in a non-PCI-capable facility, respectively [11]. The relevant prognostic role of reperfusion delays in STEMI has been demonstrated and both door-to-balloon and total ischemic time have been linked to increasing mortality [42, 45, 46]. In our study the hospital performance, evaluated in terms of appropriateness and timeliness of treatment, was found to be related with both short-term and long-term survival. This result suggests that the adherence to guidelines affects the specific phase but also the subsequent phases, pointing out the need to consider the care of the patient with AMI as an integrated pathway.

Regarding secondary prevention, our results were consistent with other studies where the effectiveness of the EB polytherapy in reducing morbidity and mortality of patients after AMI was demonstrated [27, 47, 48]. In particular, when drugs are prescribed together incremental synergistic benefits were observed [49, 50]. Despite well-established benefits, these effective secondary prevention therapies remain underutilized [51].

To date, health care systems have addressed their efforts to improve adherence to guidelines, tackling separately the single phases of the complex care process. The care of AMI patients should be considered as an integrated process that follows the patient journey [1], from the onset of symptoms to the return to a normal life. For this purpose, we identified in each phase the specific level of intervention to promote improvement in the quality of care, and we evaluated the whole process of care using a synthetic value of probability to survive that combines information from all the phases.

This approach was previously used to evaluate the care-pathway in patients with stroke [39].

Then, combining information from all the phases, we identified 18 possible care scenario that patients could have followed. When comparing people exposed to the the best and the worst process of care, we found that an optimal quality of care had a positive effect on patients’ survival. It has been shown that quality of care of patients with AMI varies with age, sex, race, geographic location, physician specialty, and hospital teaching status [6, 52, 53].

Our results did not show a gender difference in PRs, while a strong increase of PRs was observed by age classes, suggesting a higher effect of the process of care for the oldest patients. When we evaluated the subgroups of patients with chronic conditions, a slightly increase of PRs was observed for both conditions. The higher effect of the optimal continuum of care in diabetic patients was mainly due to the lower survival observed for this subgroup of patients in the acute and post-acute phases. This agrees with previous observations in other countries [54, 55]. In fact, it has been recognized that mid or long-term outcomes of AMI are poorer in diabetic than in non-diabetic patient, whereas conflicting results were found on short-term outcomes [56–59]. Moreover, our results showed a higher survival of the diabetics in the emergency phase. A possible explanation could lie in a greater responsiveness at the onset of symptoms, or a greater familiarity with the emergency systems, of patients with a more complex and severe clinical profile.

In patients with COPD, we found a lower survival in the emergency and post-acute phases, whilst no significant differences where observed in the acute phase. Although studies have reported higher long-term mortality in patients with COPD after MI, there is controversy regarding its impact on in-hospital mortality [60, 61]. Rothnie et al., showed that patients with COPD were more likely to have a delay in the diagnosis of MI and a longer time to reperfusion after STEMI, compared with non-COPD. The authors also suggested that the delay in diagnosis of MI in patients with COPD might be due to an incorrect attribution of symptoms to COPD rather than MI [62]. Furthermore, prior research has shown that β-blockers and other effective cardiac therapies are underused in patients with AMI and COPD [63].

Potential alternative explanations for our results need to be considered. In our study, we defined a synthetic measure of the multiphase quality of care by choosing and calculating indicators for the four major critical points in the continuum of care process. However, many other factors may play a role in the good prognosis in AMI patients, among them time for ambulance to arrive to patient’s house as well as pre-hospital delay times [64], referral and timely access to cardiac rehabilitation [65], healthcare provider recommendations for health behaviour change (e.g. weight management, smoking cessation) [66], unfortunately we were not able to measure factors like them, and it may represent a major limitation in the interpretation of our results.

Some other limitations of this study need to be considered. First, this study was based on data from the HIS. Despite some important advantages in collecting administrative data [67], as the large number of patients involved and the opportunity to integrate many sources of data to define and analyse MPCs, there remains questions about its accuracy, completeness and possible gaps in clinical information [68]. Secondly, in the emergency phase, the travel time was estimated from home to the nearest appropriate facility, resulting in a possible misclassification of exposure, since definitely not all patients might have been at their place of residence when STEMI occurs. However, our sensitivity analysis on the subgroups of patients aged at least 75 years, confirmed the main results.

Moreover, drug use data from our pharmaceutical database refer to the prescribed agents, but the actual levels of intake cannot be evaluated, and consequently a possible misclassification of drug utilization may have occurred. We acknowledge that prescriptions are not a measure of adherence, according to the findings and interpretation of the majority of pharmacological studies based on drug administrative registers [69].

Lastly, although several covariates were included in the models to adjust for differences in patient characteristics, we cannot exclude a residual confounding due to unmeasurable or unmeasured covariates that might affect outcome and exposure measures.

## Conclusions

In conclusion, this study demonstrated that AMI patients exposed to optimal quality of care along the continuum of the complex multiphase care management have a better 1-year survival.

Health care systems should address their efforts to improve the effectiveness of EB interventions both in the specific phases of care, and taking into account the relationships among them. Careful monitoring is required in the management of subgroups of patients with particular needs, as the oldest or those with chronic diseases.
